# Basolateral Amygdala Serotonin 2C Receptor Regulates Emotional Disorder-Related Symptoms Induced by Chronic Methamphetamine Administration

**DOI:** 10.3389/fphar.2021.627307

**Published:** 2021-02-08

**Authors:** Zhuo Wang, Chen Li, Jiuyang Ding, Yanning Li, Zhihua Zhou, Yanjun Huang, Xiaohan Wang, Haoliang Fan, Jian Huang, Yitong He, Jianwei Li, Jun Chen, Pingming Qiu

**Affiliations:** ^1^ Department of Infertility and Sexual Medicine, The Third Affiliated Hospital of Sun Yat-sen University, Guangzhou, China; ^2^ School of Forensic Medicine, Southern Medical University, Guangzhou, China; ^3^ School of Forensic Medicine, Guizhou Medical University, Guiyang, China; ^4^ Department of Neurology, The First Affiliated Hospital, School of Clinical Medicine of Guangdong Pharmaceutical University, Guangzhou, China; ^5^ Department of Neurology, Zhujiang Hospital, Southern Medical University, Guangzhou, China

**Keywords:** methamphetamine, emotional disorder, basolateral amygdala, serotonin 2C receptor, 5-HT

## Abstract

Globally, methamphetamine (MA) is the second most abused drug, with psychotic symptoms being one of the most common adverse effects. Emotional disorders induced by MA abuse have been widely reported both in human and animal models; however, the mechanisms underlying such disorders have not yet been fully elucidated. In this study, a chronic MA administration mouse model was utilized to elucidate the serotonergic pathway involved in MA-induced emotional disorders. After 4 weeks of MA administration, the animals exhibited significantly increased depressive and anxious symptoms. Molecular and morphological evidence showed that chronic MA administration reduced the expression of the 5-hydroxytryptamine (5-HT) rate-limiting enzyme, tryptophan hydroxylase 2, in the dorsal raphe and the concentrations of 5-HT and its metabolite 5-hydroxyindoleacetic acid in the basolateral amygdala (BLA) nuclei. Alterations in both 5-HT and 5-HT receptor levels occurred simultaneously in BLA; quantitative polymerase chain reaction, western blotting, and fluorescence analysis revealed that the expression of the 5-HT2C receptor (5-HT_2C_R) increased. Neuropharmacology and virus-mediated silencing strategies confirmed that targeting 5-HT_2C_R reversed the depressive and anxious behaviors induced by chronic MA administration. In the BLA, 5-HT_2C_R-positive cells co-localized with GABAergic interneurons. The inactivation of 5-HT_2C_R ameliorated impaired GABAergic inhibition and decreased BLA activation. Thus, herein, for the first time, we report that the abnormal regulation of 5-HT_2C_R is involved in the manifestation of emotional disorder-like symptoms induced by chronic MA use. Our study suggests that 5-HT_2C_R in the BLA is a promising clinical target for the treatment of MA-induced emotional disorders.

## Introduction

Methamphetamine (MA) is a highly addictive amphetamine-type stimulant (ATS) (Takayuki et al., 2018). According to the World Drug Report (United Nations, 2020), there are about 27 million global ATS abusers. In China, MA has replaced heroin as the most widely abused drug and it is also the most widely used ATS in Southeast Asia and North America. MA has a strong toxic effect on multiple organs—especially the brain—and strong withdrawal symptoms (Prakash et al., 2017; Voce et al., 2019). Chronic MA administration is accompanied by numerous emotional symptoms, such as depression, anxiety, and decreased will and activity (Bagheri et al., 2015), that increase the risk of impulsive drug use and relapse after withdrawal, thereby contributing to MA addiction. The underlying molecular mechanisms of emotional symptoms, including depression and anxiety, in chronic MA users are still unclear (Casaletto et al., 2015; Ru et al., 2019); however, revealing these could provide a theoretical basis and therapeutic targets for the reduction of and recovery from MA abuse.

The pathogenesis of depression is complicated and not yet fully understood (Gonda et al., 2019). The dysfunction of monoamine neurotransmitters—especially a decrease in the presence of 5-hydroxytryptamine (5-HT)—has been implicated in the occurrence of numerous emotional disorders, including depression (Cryan and Leonard, 2000). MA use has been shown to reduce the concentration of 5-HT in the brain (Althobaiti et al., 2016). The rate-limiting enzyme involved in brain 5-HT synthesis, tryptophan hydroxylase (TPH) 2, is mainly expressed in 5-HT neurons, originating from the raphe nucleus. The mRNA and protein expression of TPH2 is significantly decreased in the brain of a rat depression model (Angoa-Pérez et al., 2014; Chen et al., 2017). Similarly, acute and binge MA usage can affect 5-HT levels and TPH2 activity in the brain. The emotional symptoms caused by long-term drug use may be a secondary response to the changes in brain serotonergic activity.

As a neurotransmitter, 5-HT combines with the 5-HT receptor (5-HTR) to exert its regulatory function in the brain. The 5-HTR2 family is closely related to the regulation of emotion (Nic Dhonnchadha et al., 2003; Quesseveur et al., 2012). The 5-HT_2_R receptors family have a high homology, overlapping pharmacological properties, and similar second messenger signaling systems, making them attractive candidates as pharmacotherapy targets in drug abuse (Bubar and Cunningham, 2008; Cunningham and Anastasio, 2014; Howell and Cunningham, 2015). MA administration induces alterations in the expression of 5-HTRs (Mcfadden et al., 2018); however, the brain area and subtype specificity of 5-HTR2 and the causes behind MA-associated emotional disorders require further investigation. Mcfadden et al. (2018).

The basal lateral amygdala (BLA) is an integral part of the limbic system, which is responsible for the regulation of emotion (Kedo et al., 2018). Numerous studies have shown that 5-HT neurons in the dorsal raphe nucleus (DRN) exert their effects on the BLA via serotonergic regulation. In female mice exposed to 4-vinylcyclohexene, the synthesis of 5-HT in the DRN and its afferents to BLA are decreased, thereby causing impaired long-term damage in the BLA and anxiety-associated behaviors (Wang et al., 2019a). The 5-HT pathway in the DRN-BLA is also involved in the formation and recovery of fear memory, with the involvement of 5-HT_1A/2A_R signal transduction in BLA (Sengupta and Holmes, 2019). These studies suggest that the 5-HT system in the BLA plays an important role in the regulation of negative emotions.

These studies indicate that MA may affect the BLA 5-HT system. Herein, we established a chronic MA abuse model (10 mg/kg/day i. p., 4 weeks) to observe emotional symptoms and BLA serotonergic changes. The results of this study may elucidate the long-term effects of MA administration on the serotonergic system and how these contribute to the manifestation of emotional disorder-related symptoms.

## Materials and Methods

### Animals

Male C57BL/6J mice (10–12 weeks, 20–22 g) were provided by the Experimental Animal Center of Southern Medical University. The mice in each group could drink and eat freely in a standard specific pathogen-free environment, with alternating 12 h light and dark cycles (lighting interval: 7:00–19:00). All animal procedures were performed according to the National Institutes of Health guide for the care and use of animals for scientific purposes and preapproved by the Institutional Animal Care and Use Committee of Southern Medical University. To simulate chronic MA consumption, MA (purity ≥99.1%, provided by the National Institute Control of Pharmaceutical and Biological Products, Beijing, China) was administered for 4 weeks as previously described (Keshavarzi et al., 2019) (10 mg/kg i. p. daily). Ethology tests were performed after MA administration. At the end of the experiment, mice were euthanized and their brain tissues were collected for analysis. Every effort was made to minimize animal pain, suffering, and distress and reduce the number of animals used.

### Forced Swim Test

The FST was used to evaluate depressive-associated behavior in the animals (Cao et al., 2013). The experimental device was a transparent plexiglass hollow cylinder (25 cm high and 12 cm in diameter). The water level was at an approximate height of 20 cm and the water temperature was 25 ± 1 °C. During the experiment, the mice were placed gently into the water and allowed to swim freely for 6 min. Their cumulative immobility time was recorded for the last 4 min. After each experiment, the mice were removed, dried with a towel, and put back into the cage and the water in the device was changed.

### Tail Suspension Test

The tail end of the mouse was fixed with tape and hung above the ground for 6 min to suspend the head downward. The body of the mice did not contact the tail suspension instrument, except for the tail. The immobility time of mice was recorded in the last 4 min.

### Sucrose Preference Test

SPT was used to evaluate anhedonia during MA-induced depression in mice (Qin et al., 2019). In this experiment, the mice were fed in a single cage. Pure water was placed on one side of the cage and 1% sucrose solution was placed on the opposite side; the mice could freely choose between the pure water and sugar solution. The relative positions of each were randomly decided and we recorded the consumption of both. Sugar preference is expressed as a percentage of the total liquid consumption.

### Elevated Plus Maze

The EPM consisted of an open arm and closed arm. The mice were gently placed in the central area with their back to the experimenter. Video was recorded for 5 min. After the test of each mouse was completed, the device was cleaned thoroughly with 75% alcohol. The total time of open arm entry and the number of open arm entries for each mouse were counted.

### Immunohistochemistry

After anesthetization with 1% pentobarbital sodium (40 mg/kg), the heart was exposed through a thoracotomy, while the brain was extracted after perfusion. Brain tissue was immersed in 4% paraformaldehyde in saline for 24 h; subsequently, we performed gradient dehydration of the sucrose solution and immersed the brain tissue in a 30% sucrose solution. Then, we performed serial coronal sectioning (40 μm). The brain slices were rinsed with 0.1 M phosphate buffer saline (PBS) three times on a shaking table (5 min each time). A 3% bovine serum albumin (BSA) solution containing 0.5% Triton X-100 was added before drilling for 40 min. Primary antibodies (Mouse anti-GAD67, MAB5406, 1:1,000, Merck Millipore, Billerica, Mass., USA; rabbit anti-TPH2, ab184505, 1:1,000, Abcam, Cambridge, United Kingdom) were added after dilution with 0.1 M PBS containing 3% BSA and incubated in a shaker at 4 °C for 24 h. After rinsing three times with 0.1 M PBS, the corresponding fluorescent secondary antibody was added and incubated for 1 h in the dark at 25 °C. Then, the brain slices were sealed with a mounting medium (Vector Laboratories, Inc., Burlingame, CA), containing 4ʹ,6-diamidino-2-phenylindole. Images were captured under a laser confocal microscope (LSM 710; Carl Zeiss Microscopy, Thornwood, NY, United States).

### Quantitative Polymerase Chain Reaction

Total RNA was extracted from the tissue samples using TRIzol. The transcripts of the target genes in each sample were normalized with GAPDH/β-actin and expressed as a fold change using the 2^−ΔΔCt^ equation. The PCR primers used in this study are listed as follows:

1. TPH2, forward, 5′ AGC​ATT​TGG​ACG​GAG​GAA​GA 3′, reverse, 5′ TGT​ACT​CGA​CCC​TGG​GAA​TG 3′;
2. 5-HTR1A, forward, 5′CTT​TCT​ACA​TCC​CGC​TGC​TG 3′, reverse, 5′ CCC​GAC​TCT​CCA​TTC​ACA​CT 3′;
3. 5-HTR2A, forward, 5′ CTC​CTT​CAG​CTT​CCT​CCC​TC 3′, reverse, 5′ GCA​GGG​CTC​CAA​TGA​CAT​TT 3′;
4. 5-HTR2C, forward, 5′ TCG​TTC​TCA​TCG​GGT​CCT​TC 3′, reverse, 5′ CTC​ATC​ACC​CTT​CTT​GCA​GC 3′;
5. 5-HTR3, forward, 5′ GGA​CTC​CTG​AGG​ACT​TCG​AC 3′, reverse, 5′ CTA​CAG​GCG​GTC​ACC​AAT​TG 3′;
6. 5-HTR4, forward, 5′ CTG​GGC​TTA​TGG​GGA​GAT​GT 3′, reverse, 5′ GCC​ACC​AAA​GGA​GAA​GTT​GC 3′.

### Western Blotting

All brain tissues were homogenized in a protein extraction buffer (Beyotime, Shanghai, China) containing protease and phosphatase inhibitors at 4 °C for 30 min. The supernatant was collected after the lysates were centrifuged. Protein concentrations were measured using the BCA Protein Assay Kit (Beyotime, Shanghai, China). The samples were separated by sodium dodecyl sulfate-polyacrylamide gel electrophoresis and transferred to polyvinylidene difluoride membranes (Millipore, Billerica, MA, United States). The membranes were blocked in a blocking buffer at room temperature for 1 h and incubated overnight at 4 °C with anti-TPH2 (1:1,000, ab184505, Abcam), anti-5-HTR2C (1:1,000, ab137529, Abcam), and anti-GAPDH (1:5,000, ab125247, Abcam). Furthermore, the membranes were washed with a Tris-buffered saline with 0.1% Tween 20 Detergent buffer and incubated with corresponding secondary antibodies at room temperature for 1 h. The membranes were detected using electrochemiluminescence reagents (Bio-Rad, Hercules, CA, United States) and visualized using a Tanon Imaging system (Tanon, Shanghai, China). Band densities were measured using the ImageJ software and normalized to GAPDH expression. This experiment was performed in triplicate, and the representative images are presented.

### High-Performance Liquid Chromatographic With Electrochemical Detection

Monoamine transmitters in the brain homogenate were detected by HPLC. The HPLC system included a Sykam high-performance liquid chromatograph, C18 reverse-phase analytical column (DIAMONSIL, 2.1 × 100 mm, 2.5 μm), column temperature chamber, SenCell electronic flow cell (Antec, Netherlands), ADF filter (0.05 Hz), 25 μm electrode, *in situ* Ag/AgCl reference electrode, VT 03 glass carbon working electrode (3 mm), and pulse damper. The mobile phase consisted of a 15% methanol aqueous solution containing 0.74 mmol/L sodium octane sulfonate, 80 mmol/L sodium dihydrogen phosphate, 0.027 mmol/L disodium ethylenediaminetetraacetic acid, and 2 mmol/L potassium chloride adjusted to pH 3.0 with phosphoric acid. After all solutions were prepared, a 0.22-μm organic phase filter membrane was used to remove bacteria, followed by ultrasonic degassing for 30 min. The column temperature was maintained at 40 °C. Fresh standards were prepared before the experiment. The working voltage of the electrochemical detector electrode and its attenuation were 0.56 V and 200 nA, respectively.

### Stereotactic Surgery

Stereotactic surgery was conducted as previously described (Wang et al., 2019b). Briefly, after mice were anesthetized using an intraperitoneal injection of 40 mg/kg pentobarbital sodium, erythromycin eye ointment was applied to their eyes, and the hair on the top of the skull was cut off using eye scissors. Following iodine disinfection, the scalp was cut, exposing the anterior fontanelle. The position of the mouse head was adjusted to have both the front and back as well as the right and left levels of the mouse brain in the same line. The location of the BLA was: AP = −1.5 mm, ML = ±2.7 mm, and DV = −4.5 mm. The skull was drilled with a dental drill, and the drug delivery cannula (purchased from RWD) was embedded and fixed on the top of the mouse head with dental cement. It was used for drug administration and related behavior tests after 1 week of recovery.

To inject the virus, the microinjection needle (Hamilton, 5 μL, 33 g) was used to puncture to the corresponding depth and 100 nL of adeno-associated virus (AAV) was injected into the bilateral brain regions at a speed of 50 nL/min. After 5 min, the microinjection needle was slowly withdrawn to ensure that the AAV solution was fully absorbed. After suturing, kanamycin lidocaine was applied to the wound to prevent infection.

### Drug Administration

In the last week of MA administration, the relevant drug intervention experiments began. The catheter was inserted into the cannula and the infusion of the 5-HT_2A_R receptor antagonist M100907 (0.1 μmol in 100 nL) (Adrielle and Helio, 2012) and the 5-HT_2C_R antagonist sb242084 (10 nmol in 100 nL) (Ceglia et al., 2010) was performed using a microsyringe (701-RN, Hamilton, USA) connected to a microinfusion pump (KD Scientific, United Ststes) within 2 min. After the injection, the catheter was removed after 1 min. The related behavioral experiments were performed 30 min after the last administration.

### Construction of AAV-5-HT_2C_R-shRNA

Small hairpin RNA (shRNA) directed against 5-HT2CR was adapted from Anastasio et al. (2015) and verified by RT-qPCR. The shRNA sequence was synthesized and packed into rAAV-U6-shRNA (5-HT2CR)-CMV-EGFP (rAAV2) by BrainVTA (Wuhan, China). Using a similar process, rAAV-U6-shRNA (scramble)-CMV-EGFP was produced as a scrambled control. The virus (titer: 1 × 10^13^ vg/mL) was injected into the mice 2 weeks before the end of MA administration.

### Electrophysiology

After decapitation, the brain tissue was quickly removed and placed in precooled artificial cerebrospinal fluid (ACSF). After soaking for 1 min, the brain slices containing BLA tissue were transferred to an incubator in a constantly warm ACSF bath and incubated for 1 h. A glass microelectrode was used, with a tip resistance of 3.5 MQ when filled with an internal solution. We adjusted the perfusion speed to 1.5 ml/min and circulated the ACSF (124 mmol/L NaCl, 24 mmol/L NaHCO_3_, 5 mmol/L KCl, 2.4 mmol/L CaCl_2_, 21.3 mmol/L MgSO_4_, 1.2 mmol/L KH2PO_4_, and 10 mmol/L glucose, pH 7.35–7.45), continuously saturating the solution with a mixture of 95% O_2_ and 5% CO_2_. The incubated brain slices were carefully transferred to the recording tank with a pipette and fixed with a cover net. The electrode tip was adjusted to the center of the field of vision under a low-power microscope, to slowly drop the solution onto the cell and form a high-resistance sealing state. The hold current was within 100 pA. Pyramidal neurons and interneurons are distinguished by morphology and action potential (AP), as previously described (Rainnie, 1999). The SF-77 multichannel perfusion system was used for fast liquid exchange (Warner Instrument Corporation). When miniature inhibitory postsynaptic currents (mIPSCs) were recorded, magnesium-free ACSF, TTX, DNQX, and APV were added to the perfusion system to block the action potentials produced by the Na^2+^ channel and the excitatory current mediated by the N-methyl-d-aspartate (NMDA)- and α-amino-3-hydroxy-5-methyl-4-isoxazolepropionic acid-type glutamate receptors. The frequency and amplitude of mIPSCs were recorded with the membrane potential held between −70 and −80 mV. When AP was recorded, the recording mode was adjusted to the current-clamp mode. APs were evoked by a 600-ms, 100 pA depolarizing current pulse. Signals were acquired using a Multiclamp 700 b amplifier (Cellular Devices, Sunnyvale, CA, United States) and pClamp 10 (Molecular Devices, Sunnyvale, CA, United States). The recorded data were analyzed using MiniAnal software.

### Statistical Analysis

The data are expressed as mean ± standard error (SEM). SPSS (Released 2007. Version 16.0. Chicago, SPSS Inc.) was used for statistical analysis. Student’s t-test and one-way analysis of variance were used to analyze the differences between groups. Differences with *p* values <0.05 were considered statistically significant.

## Results

### Chronic MA Administration Leads to Emotional Disorder-Related Symptoms and DRN TPH2 Changes

**FIGURE 1:**
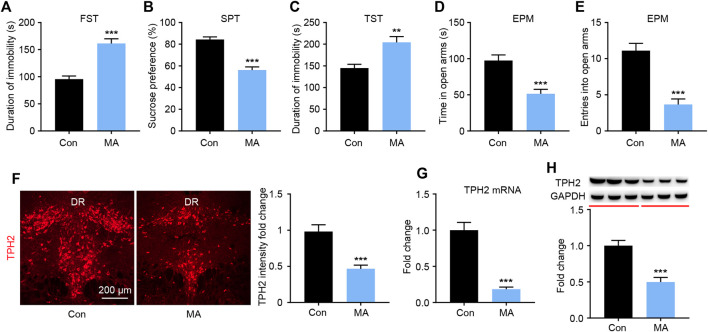
Effects of chronic MA administration on depressive symptoms and DRN 5-HT synthesis. **(A)** Immobility time during the FST. **(B)** Sucrose preference in the SPT to illuminate anhedonia. **(C)** Immobility time in the TST. **(D)** Total time exploring the open arm of elevated plus maze. **(E)** Total entries into open arm of elevated plus maze. **(F)** TPH2 immunoreactivity in dorsal raphe (DR) of the mice brain, scale bar = 200 μm. **(G, H)** TPH2 expression was quantified by qPCR and WB. DRD; dorsal part of the DR, DRV; ventral part of the DR. Data are presented as the mean ± SEM. **p < 0.01, ***p < 0.001.

After 4 weeks of administration, depression-like behaviors were measured using the FST, SPT, and TST. In the FST experiment, the immobility time in the MA group was significantly higher than that in the control group (Figure 1A). In the SPT, the sucrose preference of mice in the MA group was significantly lower than that in the control group (Figure 1B). In the TST, significantly higher mean immobility times were observed in the MA group than in the control group (Figure 1C). These three tests consistently showed the depression-like state of the animals in the MA group. To assess anxiety-like behavior, mice were placed in an EPM; those in the MA group spent significantly less time in the open arm (Figure 1D) and entered less frequently than those in the control group (Figure 1E), which indicated anxiety-like behavior. We then examined the biosynthesis of 5-HT in the DRN during the behavioral changes. The results of TPH immunofluorescence staining showed that the intensity of TPH2 immunoreactivity in the MA group was significantly lower than that in the control group (Figure 1F). The results from the qPCR and WB analysis of TPH2 showed the same tendency and verified this result (Figures 1G,H). The expression of TPH2 in the DRN was significantly lower in the MA group than in the control group and accompanied the occurrence of emotional disorder-related symptoms.

### Chronic MA Administration Affects 5-HT Metabolism in the BLA

**FIGURE 2:**
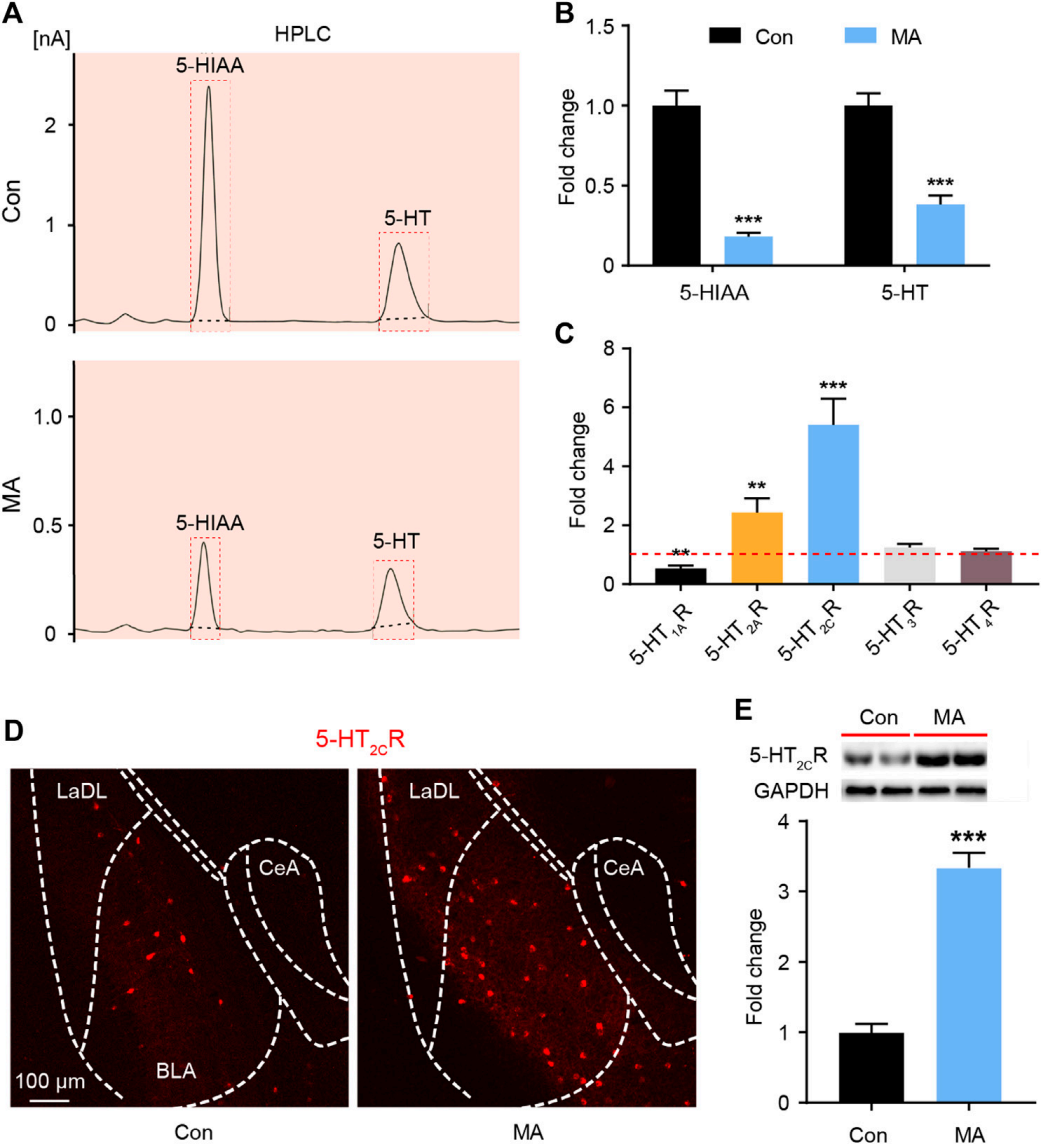
Effects ofchronic MA administration on serotonergic changes in BLA. **(A)** HPLC results of 5-HIAA and 5-HT in BLA. **(B)** Relative concentrations of 5-HIAA and 5-HT in BLA. **(C)** Relative mRNA expression of 5-HT_1A_R, 5-HT_2A_R, 5-HT_2C_R, 5-HT_3_R, and 5-HT_4_R in BLA. **(D)** 2C immunoreactivity in BLA of the mice brain, scale bar = 100 μm. **(E)** 5-HT2CR expression was quantified by WB. 5-HIAA; 5-hydroxyindole acetic acid. Data are presented as the mean ± SEM. **p < 0.01, ***p < 0.001.

It is known that DRN is the main area in the brain where 5-HT neurons are distributed. The 5-HT neurons of the DRN project to the BLA; the terminals of 5-HT neurons have been extensively marked in the BLA area (http://connectivity.brain-map.org/). BLA tissue was collected, and HPLC analyses were performed to assay serotonergic metabolism in the BLA regions of the mice. MA administration significantly decreased the levels of 5-HT and its metabolite 5-hydroxyindoleacetic acid (5-HIAA) in the BLA (Figures 2A,B). 5-HT exerts its role by binding with different receptor subtypes, so we examined the mRNA levels of the 5-HT receptors in the BLA. The qPCR results showed that the expression of 5-HT_1A_R was significantly decreased, that of 2A and 2C was significantly, and that of 5-HT_3_R and 5-HT_4_R did not significantly change after MA administration (Figure 2C). The 5-HT_2C_R immunofluorescence and WB analysis confirmed that the expression of 5-HT_2C_R in the MA group was significantly higher than that in the control group (Figures 2D,E).

### Pharmacological Inhibition of 5-HT_2C_R Alleviates Emotional Disorder-Related Behavioral Changes After Chronic MA Administration

**FIGURE 3:**
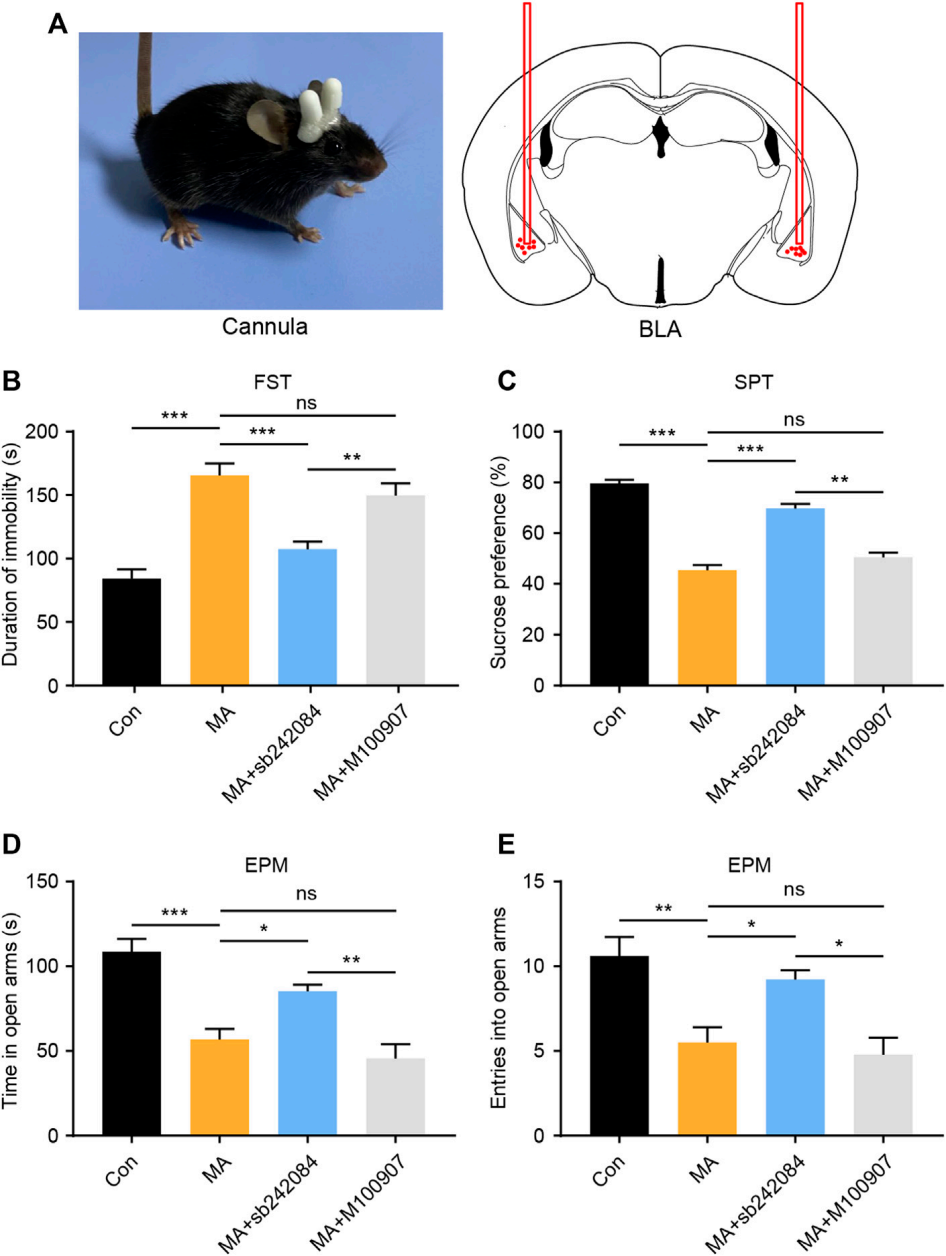
Pharmacological inhibition of 5-HT_2C_R reverses depressive and anxiety-like behaviors caused by chronic MA administration. **(A)** Schematic diagram of drug delivery strategy. **(B)** Immobility time during the FST after pharmacological intervention. **(C)** Sucrose preference in the SPT after pharmacological intervention. **(D)** Total time exploring the open arm of elevated plus maze. **(E)** Total entries into open arm of elevated plus maze. Data are presented as the mean ± SEM. *p < 0.05, **p < 0.01, ***p < 0.001.

5-HTRs are potential therapeutic targets for the treatment of emotional disorders (Amidfar and Kim, 2018). We investigated how changes in 5-HTR affected behavioral changes using a neuropharmacological strategy. During MA administration, we implanted a drug delivery cannula on the bilateral BLA and either the 5-HT_2C_R (sb242084) or the 5-HT_2A_R (M100907) antagonist was infused (Figure 3A). After 1 week of antagonist administration, we performed the behavioral tests. In the FST experiment, the immobility time of the 5-HT_2C_R antagonist group was significantly lower than that in the vehicle-treated group, whereas the 5-HT_2A_R antagonist exerted little effect (Figure 3B). In the SPT, the 5-HT_2C_R antagonist group showed higher sucrose preference than the vehicle-treated group (Figure 3C). The anxiety-like behavior was evaluated by EPM; the time spent investigating the open arm (Figure 3D) and number of entries into the open arm (Figure 3E) were significantly higher in the 5-HT_2C_R antagonist group than in the vehicle-treated group, whereas the 5-HT_2A_R antagonist treatment had little effect. These pharmacological blockade experiments show that BLA 5-HT_2C_R is a valid target against emotional symptoms when ceasing chronic MA administration.

### Effects of Virus-Mediated 5-HT_2C_R Knockdown in BLA on Behavioral Changes After Chronic MA Administration

**FIGURE 4:**
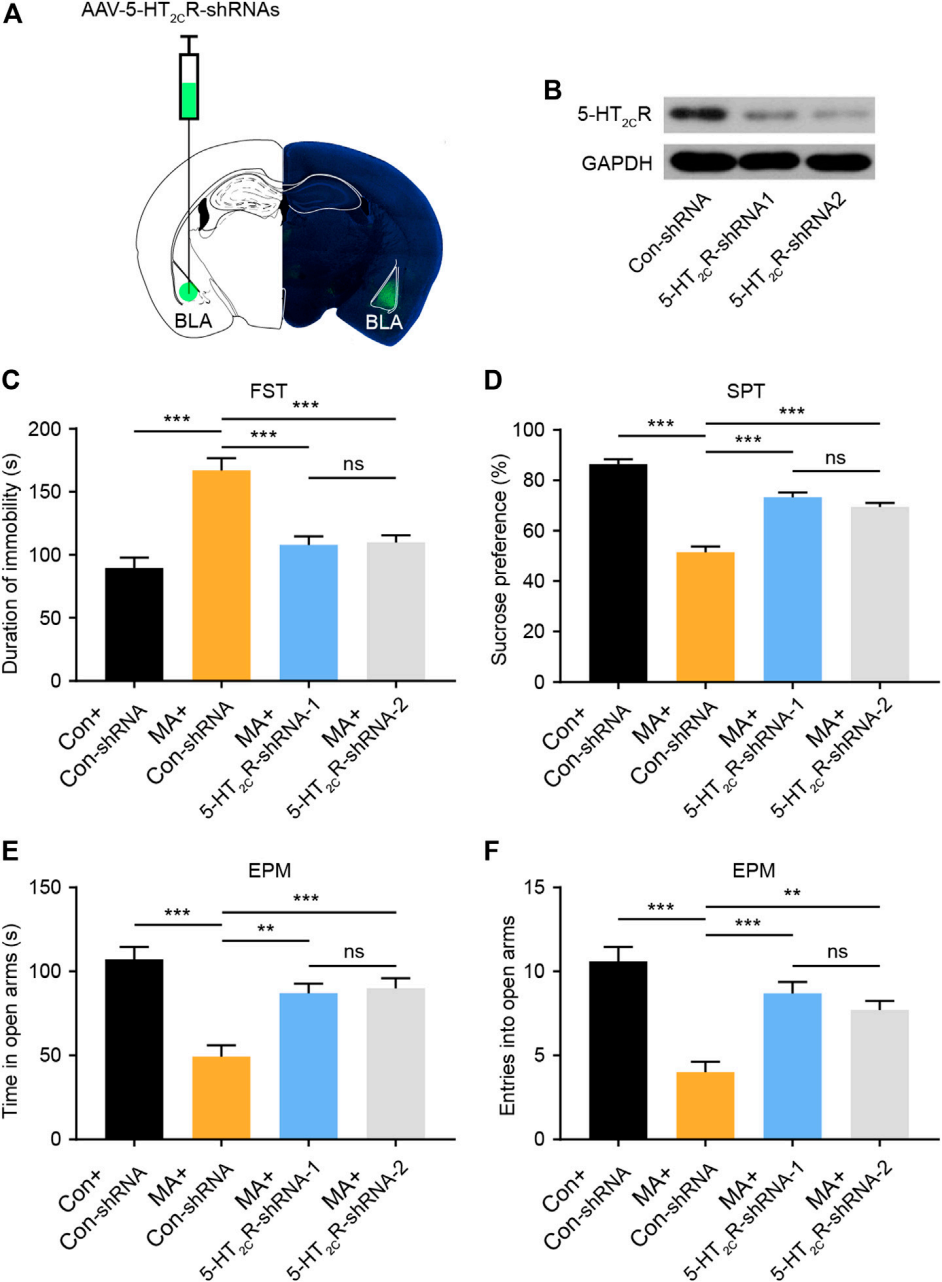
Knockdown of 5-HT_2C_R via AAV-shRNA injection reverses depressive-like behaviors caused by chronic MA administration. **(A)** Schematic of AAV vector-mediated 5-HT_2C_R knockdown in BLA. **(B)** WB results show 5-HT_2C_R expression after AAV-mediated knockdown. **(C)** Immobility time during the FST after AAV-mediated 5-HT_2C_R knockdown. **(D)** Sucrose preference in the SPT after AAV-mediated 5-HT_2C_R knockdown. **(E)** Total time exploring the open arm of elevated plus maze. **(F)** Total entries into open arm of elevated plus maze. Data are presented as the mean ± SEM. **p < 0.01, ***p < 0.001.

To confirm the role of 5-HT_2C_R in the treatment of emotional symptoms, we packed recombinant AAV2 vector-expressing shRNA against 2C (5-HT_2C_R-shRNA1/2) and nonsense shRNA as a negative control (Con-shRNA). The AAV contains a GFP tag to visualize *in vivo* expression. After AAV-5-HT_2C_R-shRNA was injected into the BLA, the virus expressed its GFP in the BLA (Figure 4A), with WB results confirming its interference efficiency (Figure 4B). We then conducted depression-screening behavioral tests. In the FST experiment, the knockdown of 5-HT_2C_R significantly increased immobility time (Figure 4C). In the SPT, the knockdown of 5-HT_2C_R reversed the decreased sucrose preference compared with that in the Con-shRNA-transfected group (Figure 4D). In the EPM test, the knockdown of 5-HT_2C_R resulted in anxiolytic-like effects on behavior in chronic MA administration mice, with significantly increased open arm stay time (Figure 4E) and number of entries (Figure 4F). These knockdown strategy results further show that BLA 5-HT_2C_R is a valid target against the emotional symptoms caused by chronic MA administration.

### 5-HT_2C_R Mediated Decreased GABA Inhibition and Increased BLA Activity During Chronic MA Administration

**FIGURE 5:**
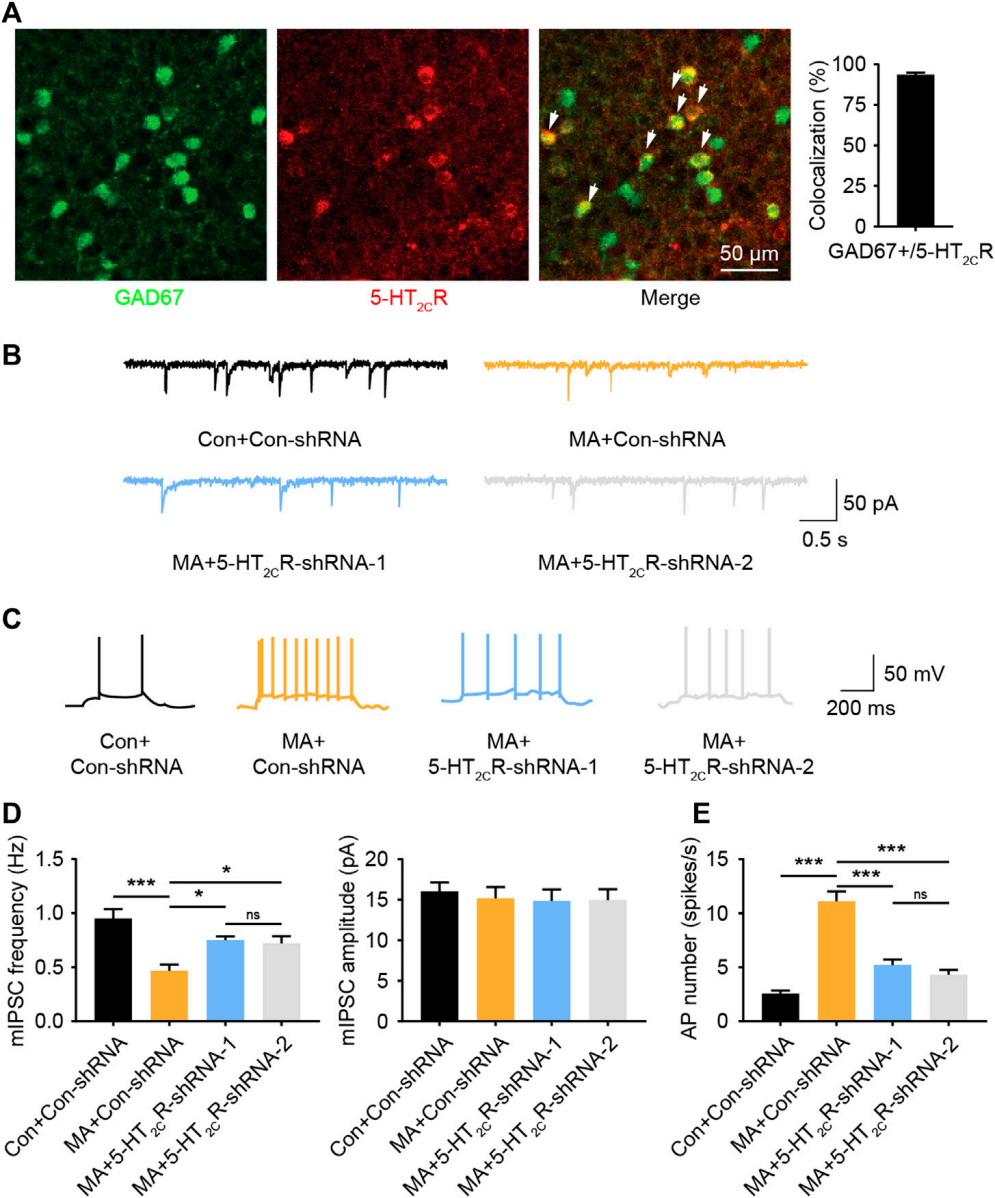
MA decreases the inhibition of BLA 5-HT_2C_R-positive interneurons and increases BLA activity. **(A)** Co-immunofluorescent staining of 5-HT_2C_R (red) and GAD67 (green) was used to reveal their colocalization, 5-HT_2C_R and GAD67 merged image is also shown (white arrow heads), scale bar = 50 μm. **(B)** Representative trace of mIPSC after AAV-mediated 5-HT_2C_R knockdown in BLA. **(C)** Representative trace of action potential after AAV-mediated 5-HT_2C_R knockdown in BLA. **(D)** Chronic MA decreased the frequency of mIPSCs; this was rescued by AAV-mediated 5-HT_2C_R knockdown, while had little effect on the mIPSC amplitude. **(E)** Chronic MA increased the frequency of action potentials; this was reversed by AAV-mediated 5-HT_2C_R knockdown. Data are presented as the mean ± SEM. *p < 0.05, ***p < 0.001.

5-HT_2C_R-positive neurons have been shown to be co-expressed with GABAergic interneurons in other brain areas (Liu et al., 2007). We performed immunofluorescent co-staining between 5-HT_2C_R and GAD67 in the BLA and found that most 5-HT_2C_R-positive neurons co-labeled with GAD67 (>93%, Figure 5A), suggesting that 5-HT_2C_R-positive neurons in the BLA are GABAergic interneurons. 5-HT_2C_R has also been found to be coupled with the GABA-A receptor (Burke et al., 2015). GABA-A is an ion channel with an inhibitory function, indicating that the function of interneurons in the local brain region of the BLA are inhibited during chronic MA administration. We detected mIPSCs in the BLA. The mIPSC frequency was lower in the MA group than in the control group; however, the amplitude remained unchanged (Figures 5B,D). After 5-HT_2C_R interference, the function of mIPSCs was partially recovered—the frequency significantly increased, which implies that there is a disinhibitory mechanism in BLA pyramidal neurons. We speculated that 5-HT_2C_R positive interneurons regulate the excitability of pyramidal neurons in the local BLA region, thereby affecting neuronal activity. Therefore, we recorded the AP of excitatory principal neurons in BLA; the frequency of BLA AP significantly increased in the MA group but decreased in the 5-HT_2C_R knockdown group (Figures 5C,E). Thus, 5-HT_2C_R-positive interneuron likely mediates neuronal activities in the BLA.

## Discussion

MA-induced emotional disorders have been widely reported in human and animal models (Glasner-Edwards and Mooney, 2014; Ru et al., 2019); however, their underlying mechanisms have not been fully elucidated. Here, we explored the potential underlying mechanisms. The results showed that chronic MA administration inhibited the expression of TPH2 in the raphe nucleus, reduced the secretion of 5-HT, and decreased the metabolism of 5-HT in the amygdala. Among the 5-HTRs, 5-HT_2C_R was upregulated in the BLA and is a potential target for therapeutic interventions of emotional disorder-related symptoms. Mechanistically, 5-HT_2C_R is mostly expressed in GABAergic neurons, exerts a disinhibitory role in BLA, inhibits the activity of GABAergic interneurons and causes an excitation imbalance of the amygdala to promote the manifestation of emotional disorder-related symptoms induced by MA administration. Here, we report, for the first time, the role of amygdala-related serotonin mechanisms in the pathogenesis of MA-induced emotional disorders.

In the brain, 5-HT regulates emotions, synaptic plasticity, learning, memory, and reward behavior (Ji and Suga, 2007; Hayes and Greenshaw, 2011; Meneses and Liy-Salmeron, 2012), as it is one of the most vital neurotransmitters. TPH2 is the rate-limiting enzyme for the synthesis of 5-HT in the brain (Al-Tikriti et al., 2012) and is essential for the biosynthesis of 5-HT in the central nervous system. MA enters the brain through the blood-brain barrier and inhibits TPH2 enzyme activity, even when exposure occurs just once (Haughey et al., 1999; Northrop and Nicole, 2015). Nevertheless, it remains unclear whether the effects of chronic MA use on 5-HT occur owing to changes in the biochemical properties of the TPH2 enzyme itself or the quantity of the enzyme. Here, we found that chronic MA administration directly inhibited the expression of TPH2 in DR-related brain regions and initiated different serotonergic molecular events related to synaptic plasticity, thereby causing subsequent behavioral changes. The DR projects onto and regulates other brain areas. In this study, we focused on the BLA, which plays a key role in the regulation of negative emotions (Kedo et al., 2018). The DR has extensive regulatory control over the BLA, suggesting that the changes in TPH2 observed in the DR could affect the metabolism of 5-HT in BLA. Previous studies have shown that this pathway participates in the regulation of multiple behaviors (Sengupta and Holmes, 2019) and is closely related to the regulation of negative emotion (Wang et al., 2019b). Therefore, we investigated local 5-HT-related metabolism in BLA. The HPLC results showed that 5-HT and 5-HIAA concentrations in the BLA were significantly decreased in MA group. The changes in the concentrations of neurotransmitters can also cause an alteration in the plasticity of the neurotransmitter-related receptors.

The interaction between 5-HT and its receptors in BLA is complex which exert diverse excitatory and inhibitory role in local microcircuit comprising multiple neuron populations (Sengupta et al., 2017). Among the receptors we tested, 5-HT_1A_R is expressed in BLA and its activation mediates the anxiolytic effect (Strauss et al., 2013), whereas 5-HT_2A_R is located on multiple neuronal types and its activation in the BLA mediates emotional symptoms (McDonald and Mascagni, 2007; Clinard et al., 2015). The increased expression of 5-HT_2C_R is associated with anxiogenic effects (Li et al., 2012), and a reduction in the expression of 5-HT_2C_R is involved in the anxiolytic effect of antidepressant drugs (Vicente and Zangrossi, 2014). 5-HT_3_R and 5-HT_4_R are also distributed in BLA and regulate emotion (Mikics et al., 2009; Chegini et al., 2014). Chronic MA administration induced generalized BLA 5-HTR changes, with 5-HT_2C_R the most affected. 5-HT_2C_R has been found to be involved in the regulation of anxiety and depression (Greenwood et al., 2012). The qPCR, immunofluorescence, and western blot results consistently confirmed that the expression of 5-HT_2C_R in the BLA was increased in the chronic MA model and that the change in the expression of 5-HT_2C_R during the chronic administration of MA was accompanied by emotional disorder-related symptoms, suggesting that 5-HT_2C_R may mediate such symptoms. Our neuropharmacological inhibition and virus-mediated knockdown strategy results confirmed 5-HT_2C_R as an appropriate therapeutic target for the treatment of chronic MA-induced emotional symptoms. As for the alteration in the expression of 5-HT_2A_R, although a previous study has suggested that targeting 5-HT_2A_R may exert beneficial therapeutic effects (Sengupta et al., 2017), our neuropharmacological intervention experiments showed that it had no significant effect on the behavioral changes. Thus, it is suspected that the mechanisms underlying the treatment of emotional symptoms are complex and that the targets and pathways in the various brain regions and etiology differ.

The amygdala neurons are mainly composed of glutamatergic and GABAergic neurons. There are multiple type of GABAergic neurons in the amygdala, which produce inhibitory effects in the local brain regions, thereby regulating the neural activities. Consistent with previous findings that 5-HT_2C_R-positive neurons are co-expressed with GABAergic inhibitory interneuron markers (Liu et al., 2007; Spoida et al., 2014), we also observed this colocalization in BLA regions. In the local brain area, 5-HT_2C_R-positive inhibitory interneurons can regulate excitatory pyramidal neurons and exert influence on the output signal of the nucleus. The GABA-A receptor, which is an ion channel with inhibitory functions, has also been found to be coupled with 5-HT_2C_R (Burke et al., 2015). The increase in 5-HT_2C_R expression can also increase the inhibitory effect of GABAergic neurons, which was confirmed by our mIPSC results. The inhibition of GABAergic interneurons in the BLA nucleus disinhibited local pyramidal neurons and caused an imbalance between excitability and inhibitory signals in the BLA. We detected the local action potential of BLA and found that the knockdown treatment strategy targeting 5-HT_2C_R successfully attenuated amygdala activation induced by chronic MA usage. This might explain the observed antidepressant effects of 5-HT_2C_R-targeted therapy.

MA users account for more than half of the drug abusers and MA abuse has become the most serious challenge in drug control in China. A study in forensic psychiatry has found that MA abusers are widely affected by emotional symptoms, such as anxiety, depression, and even suicidal ideation (Kalechstein et al., 2000). In addition, depression is the leading withdrawal symptom among MA users, and relapse is significantly associated with depressive symptoms (Zhang et al., 2014). Exploring the related pathological mechanisms and potential therapeutic targets of these emotional symptoms will not only help to improve the emotional health of MA users but also contribute to the efficacy of drug withdrawal therapy. The present study extends our understanding of the etiology and identifies the molecular targets responsible for these symptoms. In conjunction with the plasticity of serotonergic pathways that have previously been reported to be regulated by MA in BLA, we elucidated the role of 5-HT_2C_R plasticity in emotional disorders induced by chronic MA administration. Future more extensive experiments will further explore dose- and site-dependent effects of 5-HT_2C_R-targeted therapy. Furthermore, it would be interesting to explore the possibility of using 5-HT_2C_R as a treatment target in emotional disorder-related symptoms induced by chronic MA administration.

In summary, our model mimics the emotional disorder-related symptoms of chronic MA administration in MA abusers. Long-term MA administration upregulates the expression of 5-HT_2C_R in BLA, inhibits the activity of GABAergic interneurons, disinhibits local pyramidal neurons and causes abnormal BLA activity, thereby inducing emotional disorder-related symptoms. This study expands the understanding of emotional disorders among MA abusers and elucidates the potential therapeutic effect of targeting 5-HT_2C_R.

## Data Availability

The raw data supporting the conclusions of this article will be made available by the authors, without undue reservation.

## References

[B1] Adrielle V. M. Helio Z. (2012). Serotonin-2C receptors in the basolateral nucleus of the amygdala mediate the anxiogenic effect of acute imipramine and fluoxetine administration. Int. J. Neuropsychopharmacol. 15 (3), 389–400. 10.1017/S1461145711000873 21733232

[B2] Al-Tikriti M. S. Khamas W. Chebolu S. Darmani N. A. (2012). Distribution of serotonin-immunoreactive enterochromaffin cells in the gastrointestinal tract of the least shrew (*Cryptotis parva*). Int. J. Morphol. 30 (3), 916–923. 10.4067/S0717-95022012000300025

[B3] Althobaiti Y. Almalki A. Das S. Alshehri F. Sari Y. (2016). Effects of repeated high-dose methamphetamine and ceftriaxone post-treatments on tissue content of dopamine and serotonin as well as glutamate and glutamine. Neurosci. Lett. 634, 25–31. 10.1016/j.neulet.2016.09.058 27702628

[B4] Amidfar M. Kim Y. (2018). Recent developments on future antidepressant-related serotonin receptors. Curr. Pharmaceut. Des. 24 (22), 2541–2548. 10.2174/1381612824666180803111240 30073919

[B5] Anastasio N. Stutz S. Fink L. Swinford-Jackson S. Sears R. DiLeone R. (2015). Serotonin (5-HT) 5-HT2A receptor (5-HT2AR):5-HT2CR imbalance in medial prefrontal cortex associates with motor impulsivity. ACS Chem. Neurosci. 6 (7), 1248–1258. 10.1021/acschemneuro.5b00094 26120876 PMC4811199

[B6] Angoa-Pérez M. Kane M. Herrera-Mundo N. Francescutti D. Kuhn D. (2014). Effects of combined treatment with mephedrone and methamphetamine or 3,4-methylenedioxymethamphetamine on serotonin nerve endings of the hippocampus. Life Sci. 97 (1), 31–36. 10.1016/j.lfs.2013.07.015 23892197 PMC3858458

[B7] Bagheri M. Mokri A. Khosravi A. Kabir K. (2015). Effect of abstinence on depression, anxiety, and quality of life in chronic methamphetamine users in a therapeutic community. Int. J. High Risk Behav. Addict. 4 (3), e23903 10.5812/ijhrba.23903 26495258 PMC4609503

[B8] Bubar M. Cunningham K. (2008). Prospects for serotonin 5-HT2R pharmacotherapy in psychostimulant abuse. Prog. Brain Res. 172, 319–346. 10.1016/s0079-6123(08)00916-3 18772040

[B9] Burke M. V. Nocjar C. Sonneborn A. J. Mccreary A. C. Pehek E. A. (2015). Striatal serotonin 2C receptors decrease nigrostriatal dopamine release by increasing GABA-A receptor tone in the substantia nigra. J. Neurochem. 131 (4), 432–443. 10.1111/jnc.12842 PMC5329890 25073477

[B10] Cao X. Li L. Wang Q. Wu Q. Hu H. Zhang M. (2013). Astrocyte-derived ATP modulates depressive-like behaviors. Nat. Med. 19 (6), 773–777. 10.1038/nm.3162 23644515

[B11] Casaletto K. Obermeit L. Morgan E. Weber E. Franklin D. Grant I. (2015). Depression and executive dysfunction contribute to a metamemory deficit among individuals with methamphetamine use disorders. Addict. Behav. 40, 45–50. 10.1016/j.addbeh.2014.08.007 25222847 PMC4250506

[B12] Ceglia I. Carli M. Baviera M. Renoldi G. Calcagno E. Invernizzi R. W. (2010). The 5-HT receptor antagonist M100,907 prevents extracellular glutamate rising in response to NMDA receptor blockade in the mPFC. J. Neurochem. 91 (1), 189–199. 10.1111/j.1471-4159.2004.02704.x 15379899

[B13] Chegini H. Nasehi M. Zarrindast M. (2014). Differential role of the basolateral amygdala 5-HT3 and 5-HT4 serotonin receptors upon ACPA-induced anxiolytic-like behaviors and emotional memory deficit in mice. Behav. Brain Res. 261, 114–126. 10.1016/j.bbr.2013.12.007 24333573

[B14] Chen Y. Xu H. Zhu M. Liu K. Lin B. Luo R. (2017). Stress inhibits tryptophan hydroxylase expression in a rat model of depression. Oncotarget 8 (38), 63247–63257. 10.18632/oncotarget.18780 28968985 PMC5609917

[B15] Clinard C. Bader L. Sullivan M. Cooper M. (2015). Activation of 5-HT2a receptors in the basolateral amygdala promotes defeat-induced anxiety and the acquisition of conditioned defeat in Syrian hamsters. Neuropharmacology 90, 102–112. 10.1016/j.neuropharm.2014.11.016 25458113 PMC4281932

[B16] Cryan J. F. Leonard B. E. (2000). 5-HT1A and beyond: the role of serotonin and its receptors in depression and the antidepressant response. Hum. Psychopharmacol. 15 (2), 113–135. 10.1002/(SICI)1099-1077(200003)15:2<113::AID-HUP150>3.0.CO;2-W 12404340

[B17] Cunningham K. Anastasio N. (2014). Serotonin at the nexus of impulsivity and cue reactivity in cocaine addiction. Neuropharmacology, 76 Pt B, 460–478. 10.1016/j.neuropharm.2013.06.030 23850573 PMC4090081

[B18] Glasner-Edwards S. Mooney L. (2014). Methamphetamine psychosis: epidemiology and management. CNS Drugs 28 (12), 1115–1126. 10.1007/s40263-014-0209-8 25373627 PMC5027896

[B19] Gonda X. Petschner P. Eszlari N. Baksa D. Edes A. Antal P. (2019). Genetic variants in major depressive disorder: from pathophysiology to therapy. Pharmacol. Ther. 194, 22–43. 10.1016/j.pharmthera.2018.09.002 30189291

[B20] Greenwood B. N. Strong P. V. Loughridge A. B. Day H. E. Clark P. J. Mika A. (2012). 5-HT2C receptors in the basolateral amygdala and dorsal striatum are a novel target for the anxiolytic and antidepressant effects of exercise. PloS One 7 (9), e46118 10.1371/journal.pone.0046118 23049953 PMC3458100

[B22] Haughey H. M. Fleckenstein A. E. Hanson G. R. (1999). Differential regional effects of methamphetamine on the activities of tryptophan and tyrosine hydroxylase. J. Neurochem. 72 (2), 661–668. 10.1046/j.1471-4159.1999.0720661.x 9930738

[B23] Hayes D. J. Greenshaw A. J. (2011). 5-HT receptors and reward-related behaviour: a review. Neurosci. Biobehav. Rev. 35 (6), 1419–1449. 10.1016/j.neubiorev.2011.03.005 21402098

[B24] Howell L. Cunningham K. (2015). Serotonin 5-HT2 receptor interactions with dopamine function: implications for therapeutics in cocaine use disorder. Pharmacol. Rev. 67 (1), 176–197. 10.1124/pr.114.009514 25505168 PMC4279075

[B25] Ji W. Suga N. (2007). Serotonergic modulation of plasticity of the auditory cortex elicited by fear conditioning. J. Neurosci. 27 (18), 4910–4918. 10.1523/JNEUROSCI.5528-06.2007 17475799 PMC6672087

[B26] Kalechstein A. D. Newton T. F. Longshore D. Anglin M. D. Van Gorp W. G. Gawin F. H. (2000). Psychiatric comorbidity of methamphetamine dependence in a forensic sample. J. Neuropsychiatry Clin. Neurosci. 12, 480–484. 10.1176/jnp.12.4.480 11083165

[B27] Kedo O. Zilles K. Palomero-Gallagher N. Schleicher A. Mohlberg H. Bludau S. (2018). Receptor-driven, multimodal mapping of the human amygdala. Brain Struct. Funct. 223 (4), 1637–1666. 10.1007/s00429-017-1577-x 29188378

[B28] Keshavarzi S. Kermanshahi S. Karami L. Motaghinejad M. Motevalian M. Sadr S. (2019). Protective role of metformin against methamphetamine induced anxiety, depression, cognition impairment and neurodegeneration in rat: the role of CREB/BDNF and Akt/GSK3 signaling pathways. Neurotoxicology 72, 74–84. 10.1016/j.neuro.2019.02.004 30742852

[B29] Li Q. Luo T. Jiang X. Wang J. (2012). Anxiolytic effects of 5-HT₁A receptors and anxiogenic effects of 5-HT₂C receptors in the amygdala of mice. Neuropharmacology 62 (1), 474–484. 10.1016/j.neuropharm.2011.09.002 21925519 PMC3196065

[B30] Liu S. Bubar M. J. Lanfranco M. F. Hillman G. R. Cunningham K. A. (2007). Serotonin2C receptor localization in GABA neurons of the rat medial prefrontal cortex: implications for understanding the neurobiology of addiction. Neuroscience 146 (4), 1677–1688. 10.1016/j.neuroscience.2007.02.064 17467185 PMC2913252

[B31] McDonald A. Mascagni F. (2007). Neuronal localization of 5-HT type 2A receptor immunoreactivity in the rat basolateral amygdala. Neuroscience 146 (1), 306–320. 10.1016/j.neuroscience.2007.01.047 17331657 PMC1941573

[B32] Mcfadden L. M. Cordie R. Livermont T. Johansen A. (2018). Behavioral and serotonergic changes in the frontal cortex following methamphetamine self-administration. Int. J. Neuropsychopharmacol. 21 (8), 758–763. 10.1093/ijnp/pyy044 29762664 PMC6070086

[B33] Meneses A. Liy-Salmeron G. (2012). Serotonin and emotion, learning and memory. Rev. Neurosci. 23 (5-6), 543–553. 10.1515/revneuro-2012-0060 23104855

[B34] Mikics E. Vas J. Aliczki M. Halasz J. Haller J. (2009). Interactions between the anxiogenic effects of CB1 gene disruption and 5-HT3 neurotransmission. Behav. Pharmacol. 20 (3), 265–272. 10.1097/FBP.0b013e32832c70b1 19421027

[B35] Nic Dhonnchadha B. Bourin M. Hascoët M. (2003). Anxiolytic-like effects of 5-HT2 ligands on three mouse models of anxiety. Behav. Brain Res. 140, 203–214. 10.1016/s0166-4328(02)00311-x 12644293

[B36] Northrop N. A. Yamamoto B. K. (2015). Methamphetamine effects on blood-brain barrier structure and function. Front. Neurosci. 9, 69 10.3389/fnins.2015.00069 25788874 PMC4349189

[B37] Prakash M. Tangalakis K. Antonipillai J. Stojanovska L. Nurgali K. Apostolopoulos V. (2017). Methamphetamine: effects on the brain, gut and immune system. Pharmacol. Res. 120, 60–67. 10.1016/j.phrs.2017.03.009 28302577

[B38] Qin X. Wu Z. Dong J. Zeng Y. Xiong W. Liu C. (2019). Liver soluble epoxide hydrolase regulates behavioral and cellular effects of chronic stress. Cell Rep. 29 (10), 3223–3234.e6. 10.1016/j.celrep.2019.11.006 31801085

[B39] Quesseveur G. Nguyen H. Gardier A. Guiard B. (2012). 5-HT2 ligands in the treatment of anxiety and depression. Expet Opin. Invest. Drugs 21 (11), 1701–1725. 10.1517/13543784.2012.719872 22917059

[B40] Rainnie D. (1999). Serotonergic modulation of neurotransmission in the rat basolateral amygdala. J. Neurophysiol. 82 (1), 69–85. 10.1152/jn.1999.82.1.69 10400936

[B41] Ru Q. Xiong Q. Zhou M. Chen L. Tian X. Xiao H. (2019). Withdrawal from chronic treatment with methamphetamine induces anxiety and depression-like behavior in mice. Psychiatr. Res. 271, 476–483. 10.1016/j.psychres.2018.11.072 30544074

[B42] Sengupta A. Bocchio M. Bannerman D. Sharp T. Capogna M. (2017). Control of amygdala circuits by 5-HT neurons via 5-HT and glutamate cotransmission. J. Neurosci. 37 (7), 1785–1796. 10.1523/jneurosci.2238-16.2016 28087766 PMC5320609

[B43] Sengupta A. Holmes A. (2019). A discrete dorsal raphe to basal amygdala 5-HT circuit calibrates aversive memory. Neuron 103 (3), 489–505.e7. 10.1016/j.neuron.2019.05.029 31204082 PMC6687558

[B44] Spoida K. Masseck O. A. Deneris E. S. Herlitze S. (2014). Gq/5-HT2c receptor signals activate a local GABAergic inhibitory feedback circuit to modulate serotonergic firing and anxiety in mice. Proc. Natl. Acad. Sci. U.S.A. 111 (17), 6479–6484. 10.1073/pnas.1321576111 24733892 PMC4035925

[B45] Strauss C. Vicente M. Zangrossi H. (2013). Activation of 5-HT1A receptors in the rat basolateral amygdala induces both anxiolytic and antipanic-like effects. Behav. Brain Res. 246, 103–110. 10.1016/j.bbr.2013.03.005 23499701

[B61] Takayuki H. Hiroshi T. Rintaro M. Wilson D. B. (2018). Cognitivebehavioural treatment for amphetamine-type stimulants (ATS)-use disorders. Cochrane Database Syst. Rev. 12, CD011315 10.1002/14651858.CD011315.pub2 30577083 PMC6516990

[B60] United Nations (2020). World drug report. Available at: https://wdr.unodc.org/wdr2020/ (Accessed October 27, 2020).

[B46] Vicente M. Zangrossi H. (2014). Involvement of 5-HT2C and 5-HT1A receptors of the basolateral nucleus of the amygdala in the anxiolytic effect of chronic antidepressant treatment. Neuropharmacology 79, 127–135. 10.1016/j.neuropharm.2013.11.007 24275045

[B47] Voce A. Calabria B. Burns R. Castle D. Mcketin R. (2019). A systematic review of the symptom profile and course of methamphetamine-associated psychosis. Subst. Use Misuse 54 (4), 549–559. 10.1080/10826084.2018.1521430 30693832

[B48] Wang Y. Liu Y. Xiong J. Di T. Yuan Z. Wu J. (2019a). Reduced serotonin impairs long-term depression in basolateral amygdala complex and causes anxiety-like behaviors in a mouse model of perimenopause. Exp. Neurol. 321, 113030 10.1016/j.expneurol.2019.113030 31377402

[B49] Wang Z. Zeng Y. N. Yang P. Jin L. Q. Zhu X. H. (2019b). Axonal iron transport in the brain modulates anxiety-related behaviors. Nat. Chem. Biol. 15 (12), 1214–1222. 10.1038/s41589-019-0371-x 31591566

[B50] Zhang J. Xie Y. Su H. Tao J. Sun Y. Li L. (2014). Prevalence and correlates of depressive symptoms during early methamphetamine withdrawal in Han Chinese population. Drug Alcohol Depend. 142, 191–196. 10.1016/j.drugalcdep.2014.06.021 25001276

